# The longevity factor spermidine is part of a highly heritable complex erythrocyte phenotype associated with longevity

**DOI:** 10.1111/acel.14311

**Published:** 2024-09-07

**Authors:** Cameron J. Kaminsky, Jericha Mill, Viharkumar Patel, Dylan Pierce, Amelia Haj, Aaron S. Hess, Lingjun Li, Thomas Raife

**Affiliations:** ^1^ Department of Chemistry University of Wisconsin‐Madison Madison Wisconsin USA; ^2^ Department of Pathology & Laboratory Medicine University of Wisconsin‐Madison Madison Wisconsin USA; ^3^ Department of Anesthesiology University of Wisconsin‐Madison Madison Wisconsin USA; ^4^ School of Pharmacy University of Wisconsin‐Madison Madison Wisconsin USA; ^5^ Present address: Department of Pathology & Laboratory Medicine University of California‐Davis Sacramento California USA; ^6^ Present address: Harvard‐Mass General Hospital Boston Massachusetts USA

**Keywords:** association, erythrocytes, longevity, phenotype, spermidine

## Abstract

Extreme longevity in humans is known to be a heritable trait. In a well‐established twin erythrocyte metabolomics and proteomics database, we identified the longevity factor spermidine and a cluster of correlated molecules with high heritability estimates. Erythrocyte spermidine is 82% heritable and significantly correlated with 59 metabolites and 22 proteins. Thirty‐eight metabolites and 19 proteins were >20% heritable, with a mean heritability of 61% for metabolites and 49% for proteins. Correlated metabolites are concentrated in energy metabolism, redox homeostasis, and autophagy pathways. Erythrocyte mean cell volume (MCV), an established heritable trait, was consistently negatively correlated with the top 25 biomolecules most strongly correlated with spermidine, indicating that smaller MCVs are associated with higher concentrations of spermidine and correlated molecules. Previous studies have linked larger MCVs with poorer memory, cognition, and all‐cause mortality. Analysis of 432,682 unique patient records showed a linear increase in MCV with age but a significant deviation toward smaller than expected MCVs above age 86, suggesting that smaller MCVs are associated with extreme longevity. Consistent with previous reports, a subset of 78,158 unique patient records showed a significant skewing toward larger MCV values in a deceased cohort compared to an age‐matched living cohort. Our study supports the existence of a complex, heritable phenotype in erythrocytes associated with health and longevity.

## Graphical abstract

A classic twin study revealed heritable biomolecule cohorts. High spermidine levels and upregulation of autophagy/glycolysis result in increased erythrocyte lifespan.

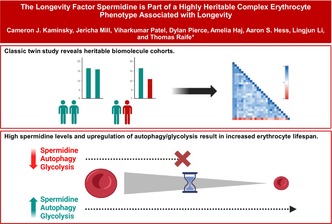

**Abbreviations**
DZ: Dizygotic
GC–MS: gas chromatography/mass spectrometry
ICC: intraclass correlation coefficient
MCV: Erythrocyte Mean Cell Volume
MS/MS: tandem mass spectrometry
MZ: Monozygotic
SMPDB: *Homo sapiens* Small Molecule Pathway Database
UHPLC: ultrahigh‐performance liquid chromatography

## 1 INTRODUCTION

The genetic contribution to longevity is of significant scientific interest. Studies to date provide convincing evidence that a component of above‐average lifespans runs in families, but much about the genetic and biochemical underpinnings of longevity remains undiscovered. Long‐lived individuals may be defined as those who experience a longer lifespan than average—currently around 76 years according to the National Center for Health Statistics. Living 100 years or more is considered extreme longevity, and an even rarer cohort of so‐called “supercentenarians” experience lifespans exceeding 110 years. While there is evidence for the heritability of lifespan in general (Finch & Tanzi, 1997), extreme longevity seems to have an even stronger genetic component. Even when adjusted for socioeconomic factors and lifestyle, siblings of centenarians are nine times more likely to also become centenarians (Perls et al., 1998), and age‐matched children of centenarians have a 62% lower risk of all‐cause mortality than children of parents with an average lifespan (Terry et al., 2004). Not surprisingly, extreme longevity is accompanied by better health and lower rates of age‐related disease. Although experiencing exceptionally long lives, supercentenarians spend an average of only 5% of their lives with an age‐related disease, like heart disease or dementia, compared to 18% in younger controls (Andersen et al., 2012).

Despite a long‐standing interest in the genetic origins of greater health span and lifespans, the few genome‐wide association studies undertaken have resulted in limited findings. Even decades after sequencing the human genome, definitive genetic causality of longevity remains undiscovered. Considering that hundreds or thousands of genetic variants may contribute to extreme longevity, the search for a genotypic profile of longevity is a daunting task. However, since longevity is a heritable phenotype, pinpointing phenotypic markers of longevity could reveal combined effects of many contributing genetic variants.

Our previous work identified highly heritable biochemical pathways and physiological phenotypes in erythrocytes (van't Erve et al., 2013, 2015; van't Erve, Doskey, et al., 2014; van't Erve, Wagner, et al., 2014; Weisenhorn et al., 2016). Since erythrocytes (by far the most numerically abundant cell type in the human body (Sender et al., 2016)) represent a metabolically active tissue, we explored in previous studies how inherited erythrocyte phenotypes may relate to inherited biological phenotypes in humans (Haj et al., 2022; Mill et al., 2022). Because blood samples are readily accessible as sources for biomarkers of health and disease, in this study, we explored heritable erythrocyte phenotypes in relation to the heritable phenotype of longevity.

Among recently identified phenotypic biomarkers of longevity is the biogenic polyamine spermidine. Spermidine is present in all eukaryotic cells and, like other polyamines, is essential for cell growth and proliferation (Larqué et al., 2007; Pegg, 2016). Spermidine is implicated in many vital biological processes, but is most often cited as a factor promoting autophagy, the process by which cells remove misfolded proteins, damaged organelles, and intracellular pathogens (Eisenberg et al., 2009; Glick et al., 2010; Hofer et al., 2022). Autophagy and energy metabolism are linked through processes like glycogen hydrolysis and physiological responses in periods of caloric restriction. Caloric restriction has consistently been shown to promote longevity across model organisms and does so by decreasing rates of glycolysis and increasing autophagic activity (Anderson & Weindruch, 2007; Das et al., 2023). Overall rates and performance of glycolysis and autophagy have been repeatedly linked to longevity, and increasing evidence suggests that autophagy declines with age as well as with many age‐related diseases. Several autophagy‐related proteins are transcriptionally downregulated in normal aging (Lipinski et al., 2010), and reduced levels of autophagy have been implicated in diseases such as osteoarthritis (Loeser et al., 2016) and cardiomyopathy (Taneike et al., 2010). As an autophagy‐associated metabolite, spermidine is considered a caloric restriction mimetic (Madeo et al., 2018), potentially providing similar benefits without reduction in caloric intake.

Like the biological process of autophagy, spermidine levels are also known to decline with age (Vivó et al., 2001). This trend is seen in some tissues like the brain (Vivó et al., 2001), but is most well characterized in blood (Scalabrino & Ferioli, 1984). Interestingly, the decline in blood spermidine concentration deviates in subjects of extreme age; a 2012 study found that although spermidine concentrations decrease in a roughly linear fashion with age, cognitively healthy nonagenarians and centenarians exhibit spermidine concentrations similar to subjects 40 years younger (Pucciarelli et al., 2012). This and other studies support the association of high spermidine levels with longevity as well as the utility of blood as a source of biomarkers for longevity. Spermidine is found in all blood components, but is present in particularly high levels in erythrocytes, accounting for roughly 70% of total spermidine measured in whole blood (Cooper et al., 1976). Erythrocytes are a unique tissue source for exploring heritable biochemical pathways. They are easily isolated from whole blood, are metabolically active, and have a relatively long and stable lifespan compared to other blood constituents (Barcellini). As evidence of their in vivo stability, erythrocytes exhibit a number of heritable metabolic pathways (Gilroy et al., 1980; Stolwijk et al., 2021; van't Erve et al., 2013, 2015; van't Erve, Doskey, et al., 2014; Weisenhorn et al., 2016). By contrast, plasma and serum contain heritable components whose cell source cannot be isolated and from which the characteristics of metabolic pathways can only be inferred. Erythrocytes often express metabolic markers that are significantly more informative compared to whole blood (Sánchez et al., 2023). The numerous leukocyte populations in blood, and their phenotypic variability in response to various stimuli, are likely reasons why there are no published reports on the heritability of biochemical pathways in leukocytes. Because of their abundance, accessibility, and metabolic stability, erythrocytes are an ideal model for the study of heritable metabolic traits.

Spermidine is synthesized endogenously from the amino acids arginine, ornithine, and methionine (Minois et al., 2011). In vivo concentration is also modified through diet. Exogenous supplementation in mice and other model organisms has been demonstrated to improve health and promote longevity in an autophagy‐dependent manner (Eisenberg et al., 2016). Across multiple Asian countries, dietary levels of spermidine correlate with life expectancy (Binh et al., 2010), and higher spermidine dietary content was correlated with markedly lower mortality rates in an Italian population (Kiechl et al., 2018). Spermidine is also produced in appreciable quantities by the gut microbiome (Tofalo et al., 2019).

We observed that blood concentrations of spermidine and a cluster of metabolites and proteins potentially linked to autophagy are highly heritable. Interestingly, this cluster of molecules is negatively correlated with erythrocyte mean corpuscular volume (MCV), which is also a heritable trait. MCV itself was observed to display an age‐related relationship to all‐cause mortality. MCV and the associated cluster of biomolecules appear to comprise a complex heritable erythrocyte phenotype associated with longevity.

## 2 EXPERIMENTAL METHODS

### 2.1 Erythrocyte multi‐omics twin study

This study utilized a previously reported twin study database comprising metabolomics and proteomics of erythrocytes in adult twins (Haj et al., 2022; Stolwijk et al., 2021; van't Erve et al., 2013, 2015; van't Erve, Doskey, et al., 2014; Weisenhorn et al., 2016). The study was approved by the Human Subjects office of The University of Iowa Carver College of Medicine. Written informed consent was obtained from all participating subjects. Subjects qualified for participation by meeting criteria for autologous blood donation according to standard operating procedures of The University of Iowa DeGowin Blood Center. Five dizygotic (DZ) and 13 monozygotic (MZ) twin pairs participated in this study, but twin pairs were not required to donate samples concurrently. Standard health history and demographic information were obtained at the time of enrollment and informed consent.

Blood sampling and proteomic/metabolomic analyses were described previously (Weisenhorn et al., 2016). Briefly, whole blood collected from study participants was centrifuged, followed by the removal of the plasma and buffy coat. Blood was processed according to standard operating procedures into a leukocyte‐reduced RBC unit. Aliquots of erythrocytes were pipetted from the center of the red cell mass, washed, lysed in nanopure water, mixed, and stored at −80°C until analysis. DNA obtained from leukocyte reduction filters was used for zygosity testing.

Samples were prepared as previously described for metabolomics analysis (Evans et al., 2009; van't Erve, Doskey, et al., 2014) using both ultrahigh‐performance liquid chromatography/tandem mass spectrometry (UHPLC–MS/MS), optimized both for acidic and basic species, and gas chromatography/mass spectrometry (GC–MS). Samples were also prepared as previously described for proteomics analysis via LC–MS/MS. Data analysis was performed as previously described using MaxQuant software version 1.5.2.8 (Cox & Mann, 2008) and the Andromeda search engine. Pearson correlations were calculated among all proteins and metabolites using Perseus software. The one‐way model of intraclass correlation coefficient (ICC) was used to determine the similarity of a measure in a twin pair: ICC = (MS_between_ − MS_within_)/(MS_between_ + MS_within_), where MS_between_ is the estimate of the mean‐square variance between all twin‐pairs and MS_within_ is the estimate of the mean‐square variance within the sets of pairs in that group. From the ICC values, heritability was estimated using the method derived by Newman et al., *h* ^2^ = (ICC_MZ_ − ICC_DZ_)/(1 − ICC_DZ_) (Newman et al., 1937). Heritability estimates were calculated for all data elements in which data were extant for at least three twin pairs in the MZ and the DZ cohort. Processed data containing all heritability estimates and twin pair data are openly available through Harvard Dataverse as “RBC Twin Study Metabolite and Protein Heritability Raw and Processed Data” (Raife).

The metabolomics data were uploaded to the National Metabolomics Data Repository at project ID PR001912 (Raife). Proteomics data were uploaded to the Center for Computational Mass Spectrometry MassIVE database project ID MSV000093812 (Raife).

### 2.2 Metabolite pathway enrichment and gene ontology analysis

To visualize pathways of interest, metabolite pathway enrichment analysis was performed using MetaboAnalyst 5.0 Pathway Analysis tool (Pang et al., 2021). Metabolites significantly correlated with spermidine were entered as compound names. Pathway analysis parameters were as follows: Only use metabolite sets containing at least two entries; reference metabolome: use all compounds in the selected pathway library. The *Homo sapiens* Small Molecule Pathway Database (SMPDB) pathway library was used as reference. Enriched pathways with FDR ≤0.1 were considered significant. Gene Ontology analysis was performed using ShinyGO 0.77, with an FDR cut‐off of 0.5 (Ge et al., 2020).

### 2.3 Analyses of erythrocyte MCV in relation to age and living status

To explore the relationship between erythrocyte MCV and longevity, data were obtained from UW Health Enterprise Analytics after designation of exempt subject status by the UW‐Madison Minimal Risk IRB. The UW Health Enterprise Analytics database contains all medical record data from UW Health patients since the implementation of the Epic electronic medical record system in 2003. The query called for the most recent available MCV and matching age for unique medical record numbers. The MCV dataset is openly available through Harvard Dataverse as “Erythrocyte Mean Cell Volume vs Living Status”(Raife).

To assess the relationship between MCV and age, a random 10% of all MCV data points (total *N* = 432,682) were selected and plotted by age. Although the relationship between mean MCV and age was linear for most of the age span, there appeared to be a significant change in the slope of the line between 85 and 87 years of age. To confirm this observation, a piecewise linear spline model with a single knot was fitted; the knot location was adjusted until the Akaike information criterion was minimized, indicating best possible fit (Akaike, 1974). After identification of the optimized spline model, that model was applied to the remaining 90% of the data to test for significance.

To characterize the relationship between MCV and all‐cause mortality, every unique patient in the Enterprise Analytics database described above was separated by last known vital status (alive versus dead), then stratified by decade of age at time of death (<21, 21–30, 31–40, etc.). The most recent MCV measurement was abstracted for each patient. The number of patients with each MCV value was then plotted in a histogram by vital status and decade of age. The distribution of MCV values between the living and the dead, stratified by decade of age, were compared using the Kolmogorov–Smirnov test (Naaman, 2021), using the Benjamini–Hochberg procedure to control for multiple comparisons (Benjamini, 2010).

## 3 RESULTS

### 3.1 Heritability of metabolic pathways in erythrocytes

**FIGURE 1:**
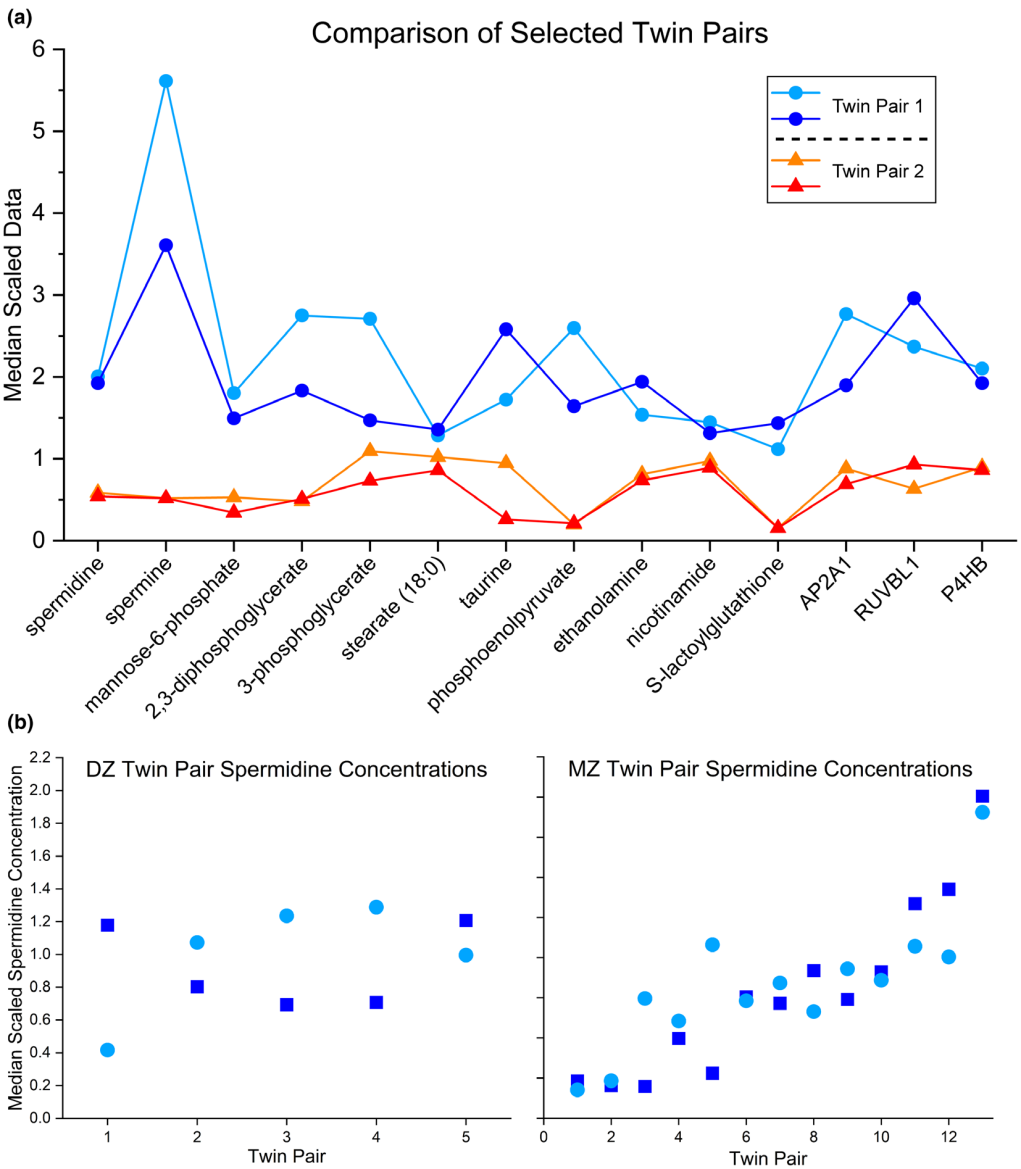
(a) Comparison of erythrocyte concentrations of biomolecules highly correlated with spermidine in selected monozygotic twin pairs. The concordance in intrapair concentrations and biological variability between pairs is illustrated. Intrapair concordance and between pair differences are the basis for calculated heritability estimates. Concentration values are displayed scaled to the data median. (b) Comparison of MZ and DZ twin pair spermidine levels ordered by mean concentration. When plotted in this fashion, it becomes clear that while DZ twin pairs have little concordance with respect to erythrocyte spermidine concentration, MZ twin pairs have much more closely related spermidine levels. Such a phenomenon is the basis for a high heritability estimate.

Previous studies identified highly heritable major metabolic pathways in erythrocytes, including energy metabolism and redox homeostasis (Gilroy et al., 1980; Stolwijk et al., 2021; van't Erve et al., 2013, 2015; van't Erve, Doskey, et al., 2014; Weisenhorn et al., 2016). Figure 1a illustrates intrapair concordance and biological variability of selected analytes in exemplary MZ twin pairs. Of 329 metabolites in the database, 45% were at least 30% heritable. Heritability estimates were calculated for all data elements in which data were extant for at least three twin pairs in the MZ and the DZ cohort.

### 3.2 Spermidine concentration is highly heritable in erythrocytes

Erythrocyte spermidine concentration has an 82% heritability estimate (data were available for all 36 subjects). Figure 1b compares DZ and MZ twin pair spermidine concentrations showing biological variability and tighter concordance in MZ twin pairs. Although spermine data were too few to calculate heritability, its strong correlation with spermidine (*R* = 0.736; *p* = 3.22 E‐07) suggests that it is probably heritable as well.

### 3.3 Spermidine concentration is positively correlated with heritable molecules in energy metabolism, redox, and autophagy pathways

**FIGURE 2:**
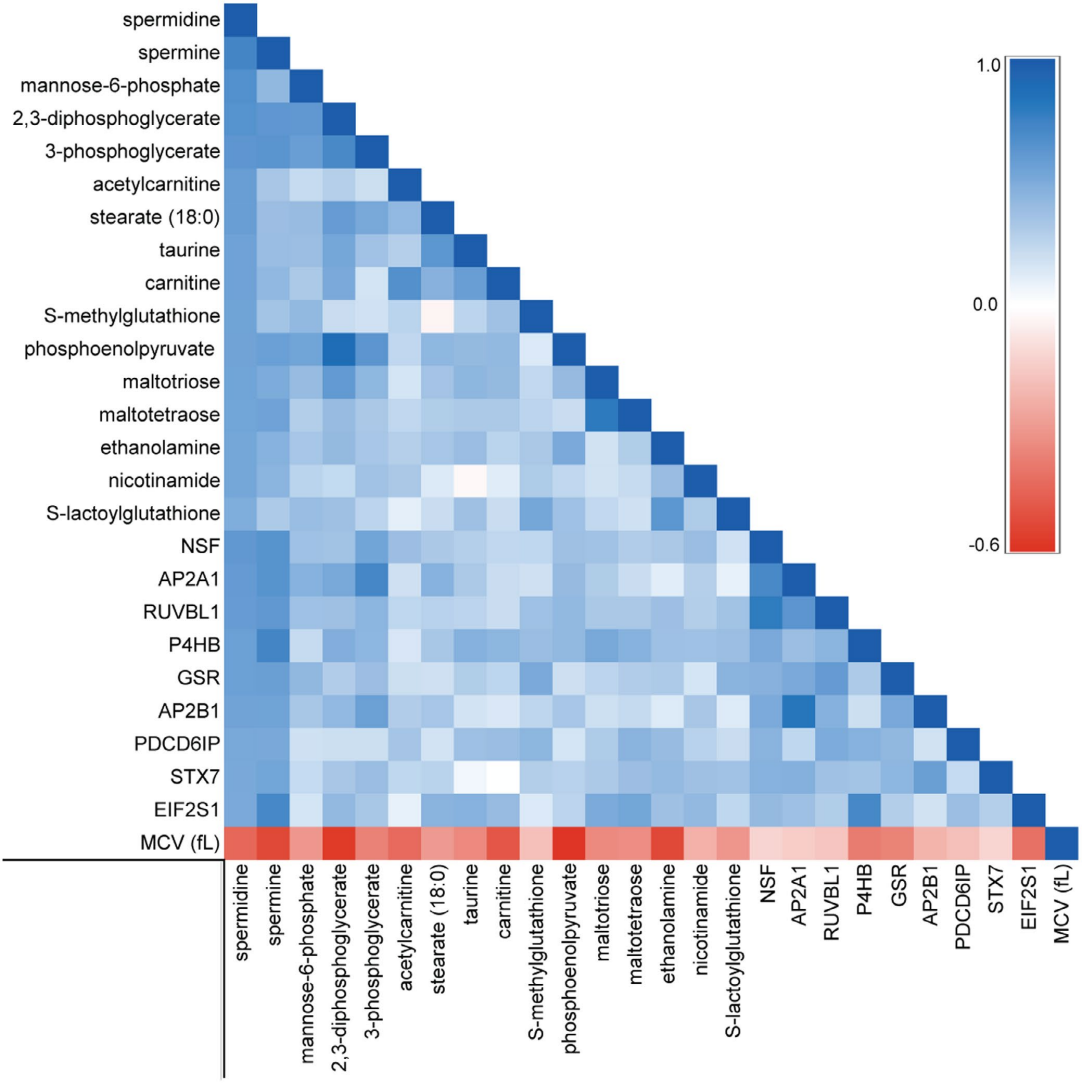
Correlation matrix of MCV and the top 25 biomolecules most highly correlated with spermidine. The consistent positive correlations between biomolecules and negative correlations with MCV indicate that regulation and inheritance of these phenotypic components are shared, supporting a longevity‐related complex phenotype.

To identify possible novel relationships among the identified molecules and spermidine, Pearson correlations were calculated for all erythrocyte metabolites, proteins, and metadata elements. The metabolites and proteins significantly positively or negatively correlated with spermidine are listed in Tables S1 and S2, respectively. Prominent among the most strongly positively correlated molecules are metabolites and proteins involved in energy metabolism, redox homeostasis, and autophagy (Figure 2).

Spermidine correlated significantly with intermediate metabolites in the glycolysis and gluconeogenesis pathways, including pyruvate, 3‐phosphoglycerate, phosphoenolpyruvate, 2,3‐diphosphoglycerate, and dihydroxyacetone phosphate. Spermidine and the downstream metabolite spermine are additionally known to play a role in the antioxidant glutathione pathway. Three metabolites belonging to this pathway—glutathione, glycine, and glutamate—are also significantly correlated with spermidine. Metabolites in both glycolysis/gluconeogenesis and glutathione metabolism were also highly heritable (Table S1). Nine of the 15 most strongly correlated metabolites were nominally heritable with a mean heritability estimate of 69%.

One of the most highly correlated metabolites, mannose‐6‐phosphate (*R* = 0.678; *p* = 5.58E‐06), is part of the fructose and mannose metabolism pathway, of which two other highly correlated metabolites are a part (guanosine 5′‐diphospho‐fucose and dihydroxyacetone phosphate). Mannose‐6‐phosphate levels are highly heritable (64%). Mannose‐6‐phosphate tags lysosomal enzymes traveling through the Golgi apparatus and directs them to the late endosome, an autophagy process that is essential for degradation of proteins. The strong positive correlation between these autophagy‐related molecules and their heritability are important novel observations of this study.

**FIGURE 3:**
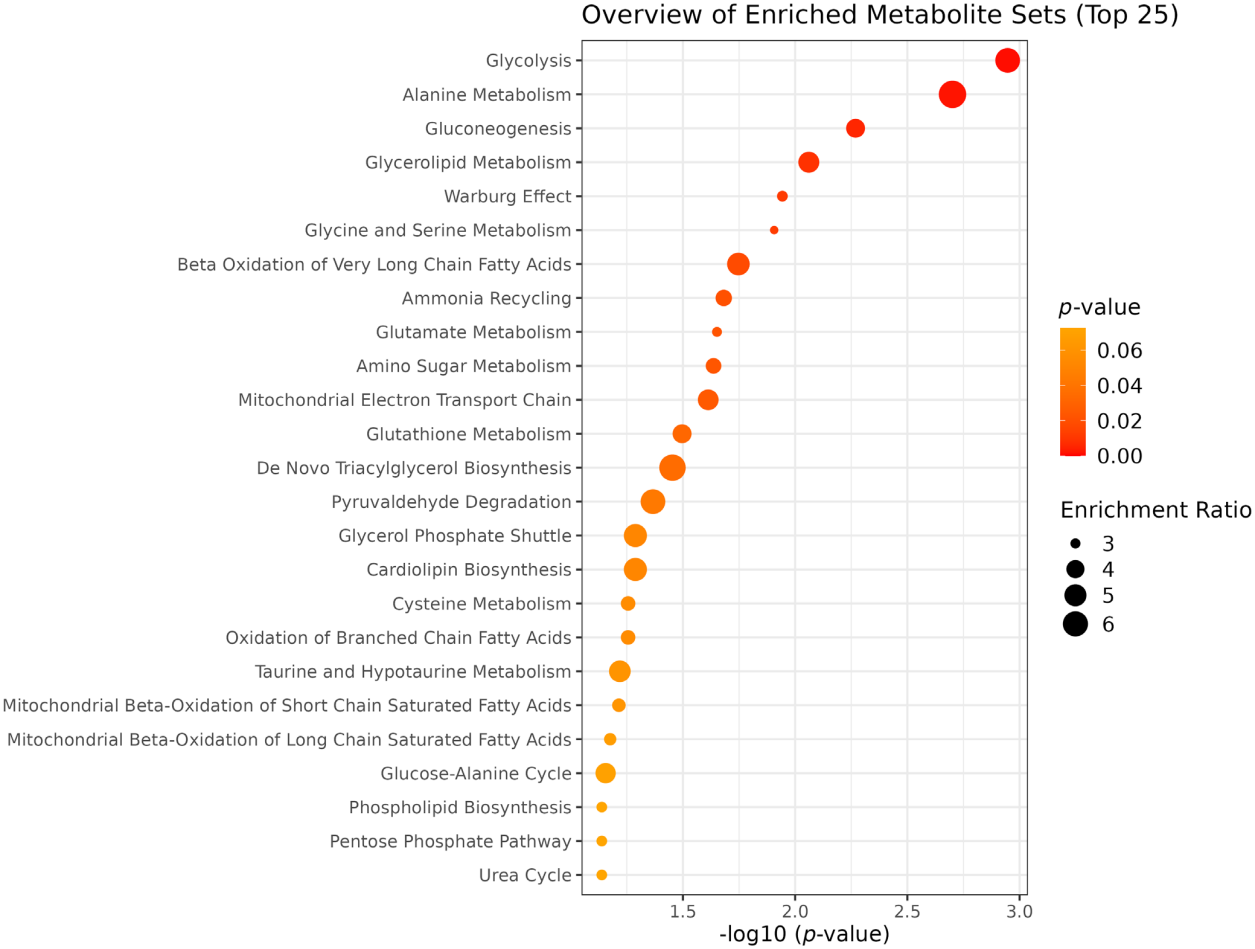
Pathway enrichment analysis of metabolites having Pearson correlation coefficients with spermidine ≥0.35. Many pathways showed significant enrichment when analyzing the metabolites most closely correlated with spermidine. Notably, glycolysis was the most significantly enriched, with five metabolites (pyruvate, 3‐phosphoglycerate, phosphoenolpyruvate, 2,3‐diphosphoglycerate, and dihydroxyacetone phosphate) correlated with spermidine.

To identify metabolic pathways among molecules highly correlated with spermidine, pathway enrichment analyses were performed using MetaboAnalyst on metabolites having Pearson correlation coefficients with spermidine ≥0.35 (Figure 3). These analytes are compared against the complete *Homo Sapiens* SMPDB to identify pathways highly represented in this subset of selected metabolites. As expected, energy metabolism and redox metabolism pathways were identified. Other pathways represented among the correlated metabolites include amino acid metabolism.

**FIGURE 4:**
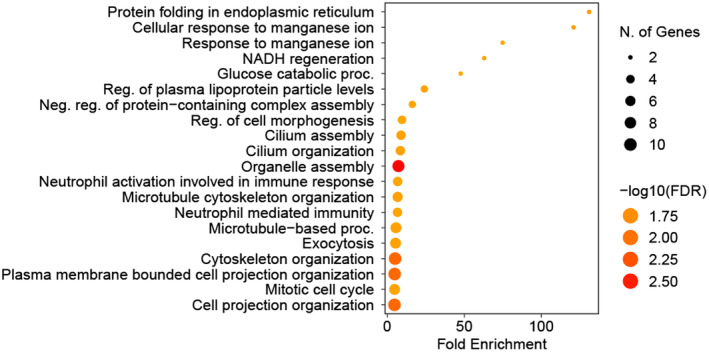
Gene Ontology analysis of proteins having Pearson correlation coefficients with spermidine ≥0.35. Focusing specifically on biological processes, several annotations were significantly enriched when analyzing correlated proteins. Of note, protein folding and trafficking through the endoplasmic reticulum as well as other protein organization and localization processes have high levels of enrichment and genes enriched. There are also multiple pathways (glucose metabolism, NADH regeneration) dealing with regulation of cellular respiration and energy metabolism pathways. Manganese response pathways are highly enriched as well and are implicated in (among other things) autophagy regulation and promotion.

Several autophagy‐related proteins are highly correlated with spermidine and highly heritable (Table S2). A Gene Ontology analysis of proteins correlated *R* ≥ 0.40 with spermidine identifies proteins involved in both intracellular protein transport and vesicle‐mediated transport (Figure 4). Among these are NSF, which is required for vesicle fusion, and STX7, which is involved in autophagosome‐lysosome fusion. GANAB plays a role in protein quality control in the endoplasmic reticulum, and RNH1 plays a role in reactive oxygen species homeostasis. The mean heritability estimate of these four molecules is 46%. Among the correlated proteins involved in intracellular protein transport (XPO7, HSPA5, CHMP4A, EHD1, NSF, STX7), the latter three were ≥30% heritable. Among proteins involved in vesicle‐mediated transport (EHD1, CHMP4A, NSF, ABCC4, DIAPH1, PA2G4, and STX7), ABCC4 and PA2G4 were additionally ≥30% heritable. While spermidine is known to induce autophagy by inhibiting the acetyltransferase EP300 (Pietrocola et al., 2015), the associations we observed suggest possible novel relationships—direct or indirect—between spermidine and other proteins implicated in autophagy‐associated processes.

Included in this network is an observed enrichment in manganese response‐associated proteins. This connection is not entirely surprising, as relatively recent work strongly implicates a complex relationship between manganese toxicity and autophagy (Ma et al., 2020; Yan & Xu, 2020).

### 3.4 Erythrocyte MCV is negatively correlated with molecules positively correlated with spermidine

Among metadata in the twin erythrocyte database, MCV was found to be strongly negatively correlated with spermidine with a Pearson correlation of −0.44. As might be expected, MCV was also negatively correlated with many of the same energy metabolites and autophagy‐related proteins that were positively correlated with spermidine (Figure 2). The consistent negative correlations between MCV and spermidine‐related molecules suggested the hypothesis that MCV is part of a complex erythrocyte phenotype associated with longevity. MCV has long been known to be highly heritable, and smaller MCVs are associated with longer circulating erythrocyte lifespan (Evans et al., 1999; Lin et al., 2007; van't Erve et al., 2015; Whitfield et al., 1985).

### 3.5 Nonlinear trends in MCV versus age

**FIGURE 5:**
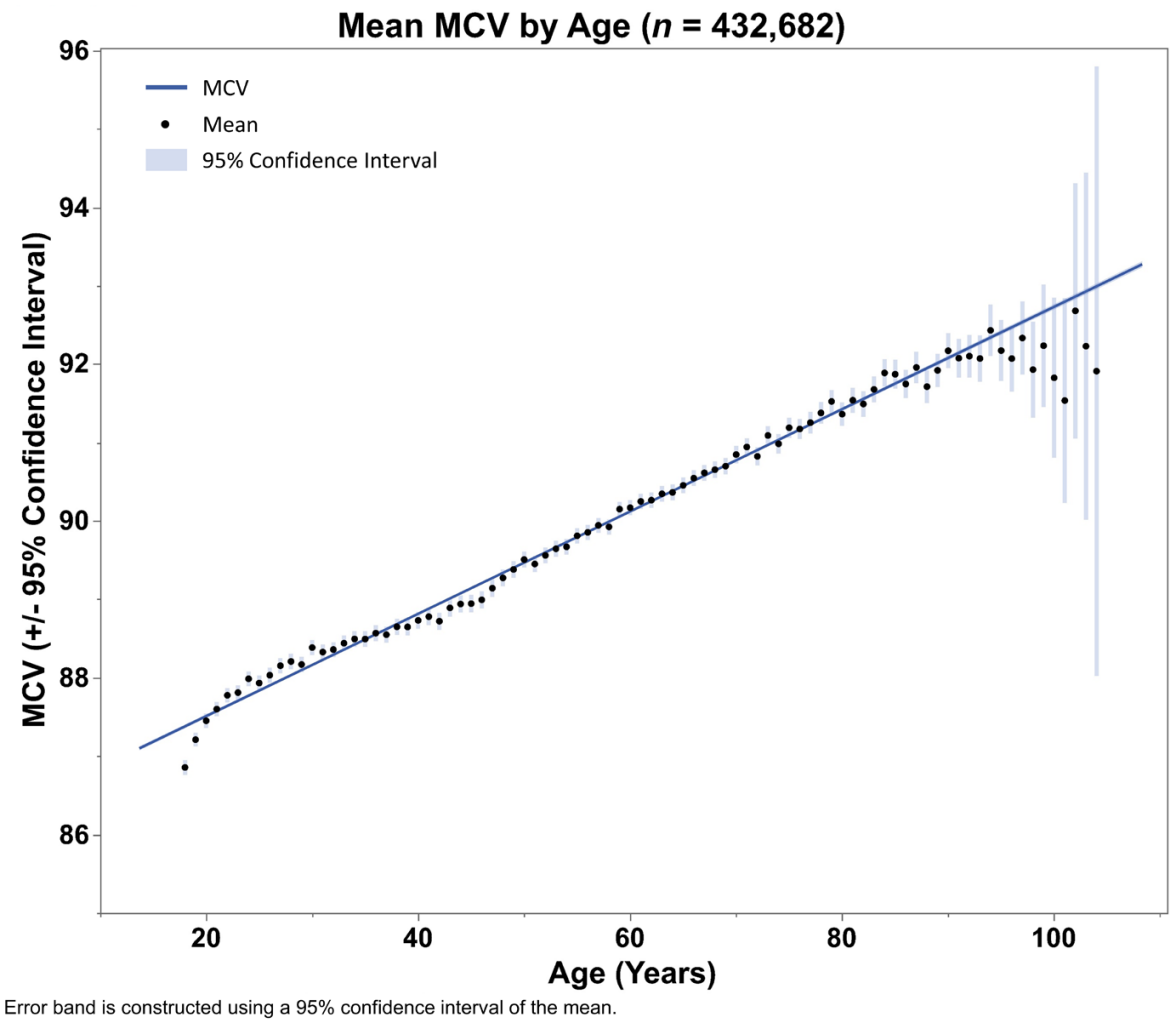
Average MCV plotted for patients of each age. Data from 433,249 unique patients reveal mean MCV values increasing with age, as expected, but skews toward smaller values at advanced age (>86 years). Using a piecewise linear regression model trained on a 10% subset, the best fit to the data was determined by minimization of the Akaike information criterion, identifying a significant downward inflection at age >86 years. The model was then validated on the remaining 90% of the data (*n* = 389,924), verifying a significant inflection in the relationship between age and mean MCV above 86 years of age (*p* for the change in slope <0.001).

Consistent with established trends (Gamaldo et al., 2011; Yip et al., 1984; Zierk et al., 2020), in a database of 432,682 unique subjects drawn from UW Health medical records, we observed that MCV is highly correlated with age and increases throughout life. However, further analysis reveals a novel trend in which MCV skews toward smaller values in old age (Figure 5). Beyond 86 years of age, the relationship between MCV and age demonstrates a significant deviation from the overall regression line. This significant departure in long‐lived subjects from the otherwise linear increase in MCV with age supports the hypothesis of a relationship between extreme longevity and smaller MCV. This observation is concordant with the observation of inverse correlations between MCV and molecules associated with autophagy, energy metabolism, and redox homeostasis (Figure 2).

### 3.6 Higher MCV is associated with mortality

**FIGURE 6:**
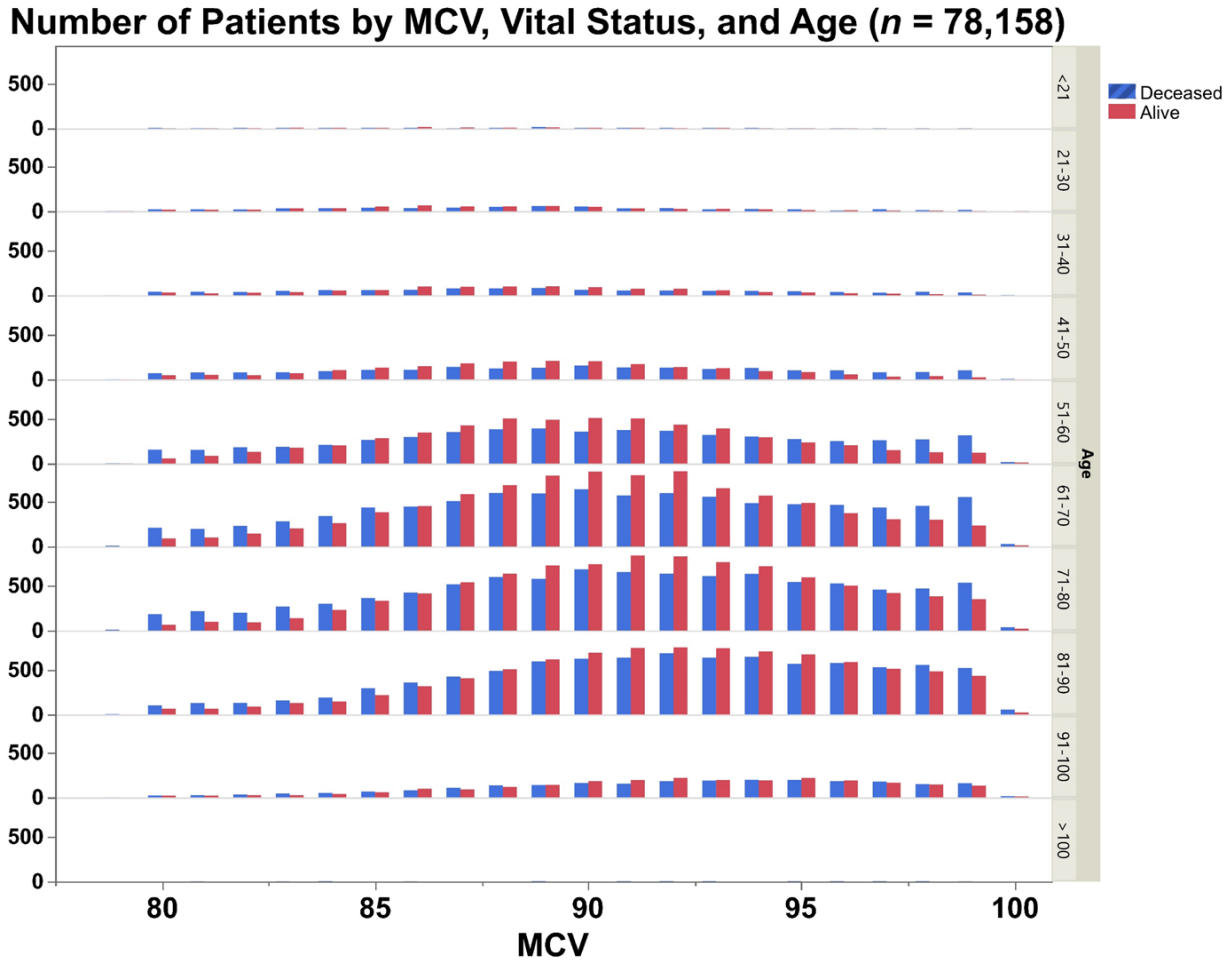
Plotted data of the most recent MCV value for patients separated into alive or deceased status. Data were stratified based on subject age; then, Kolmogorov–Smirnov tests were conducted on each age bin using the Benjamini–Hochberg false discovery rate for multiple comparisons. In the six middle bins (age bins spanning 31–90 years old), there is a significant difference in distribution of alive/deceased patient MCVs (FDR <0.05 for all). In all six of these bins, the distribution of deceased patient MCVs is flatter and more right skewed. Sample sizes for the two oldest and two youngest bins appear insufficient to establish statistically significant differences.

The relationship between MCV and mortality was explored in an age‐stratified analysis of UW Health patient medical records in which alive/deceased status was queried in addition to MCV and age (Figure 6). In this analysis, the deceased cohort (all‐cause mortality) showed a statistically significant difference in MCV across all age groups between 30 and 90 years of age as determined by a Kolmogorov–Smirnov test. The analysis revealed a higher average MCV, and a right‐skewed distribution (toward higher MCVs) in the deceased compared to the alive cohort. Similar to a previous study (Yoon et al., 2016), across all ages from the 4th decade to the 10th, a higher MCV is associated with higher rates of all‐cause mortality. Higher mortality was also observed across age groups in the lowest ranges of MCV, but with much lower confidence than at the high MCV range. This may represent deaths associated with infections, inflammation, nutritional status, or other factors associated with relative microcytosis, as was previously reported (DeLoughery, 2014; Honda et al., 2020).

## 4 DISCUSSION

While the heritability of longevity is well established through numerous twin and family studies, few longevity‐associated genes have been identified. In this study, we interrogated an established twin study database of erythrocyte proteins, metabolites, and metadata revealing that the longevity factor spermidine and a cluster of metabolites and proteins involved in autophagy, energy metabolism, and redox homeostasis are positively correlated and highly heritable. These biochemical pathways are known to be important in sustaining health and longer lifespans.

Deficiencies are implicated in fatal conditions such as neurodegenerative disease (Patel et al., 2023; Wong et al., 2020). We further observed that this cluster of biomolecules is negatively correlated with MCV, suggesting a heritable longevity‐related erythrocyte phenotype.

Much existing work regarding changes in glycolysis and energy metabolism in aging populations focuses on specific disease states, such as Alzheimer's disease (Lista et al., 2023; Mill & Li, 2022; Patel et al., 2023; Shippy et al., 2020). The cluster of associated molecules identified in this work implicates regulation of glycolysis as a process central to aging and suggests that glycolytic and autophagic pathways are inextricably linked to healthy aging, even in the absence of age‐related disease. Although spermidine is most often referenced in relation to autophagy, it is also linked to energy metabolism through spermidine/spermine N1acetyltransferase (SSAT). SSAT is the rate‐limiting enzyme in polyamine catabolism, a process in which spermidine and spermine are acetylated via acetyl‐coenzyme A (CoA), an end product of both glycolysis and fatty acid oxidation (Casero Jr & Pegg, 1993). A further relation to energy metabolism is the correlation between spermidine and the ketone 3‐hydroxybutyrate, an energy substrate in brain whose concentration is markedly diminished in both blood and brain in Alzheimer's disease (Shippy et al., 2020). These associations support the presented evidence that changes in polyamine flux, both biosynthesis and degradation, are linked to energy metabolism. This conclusion is also consistent with abundant evidence of longevity promotion through caloric restriction, which promotes autophagy and elevates 3‐hydroxybutyrate concentrations.

Our data indicate that spermidine has a high degree of heritability, ranking in the 97th percentile of all metabolites identified in this twin study. There is likely a series of genetic components responsible for this inheritance, but it is also feasible that the gut microbiome—known to be heritable—plays a role through its contribution of spermidine to the body's own pool (Badal et al., 2020; Benson et al., 2010; Grieneisen et al., 2021). This link is speculative, but the possibility is consistent with current knowledge regarding the microbiome.

The association between dietary spermidine content and longevity is well documented in both experimental and epidemiological studies (Binh et al., 2010; Kiechl et al., 2018; Madeo et al., 2018; Schroeder et al., 2021). However, plasma and whole blood concentrations of spermidine are not consistently correlated with spermidine supplementation in humans (Senekowitsch et al., 2023; Soda et al., 2009). In fact, some studies suggest that spermidine dietary content is more closely associated with spermine concentration in plasma, and that spermine concentration in food is more strongly correlated with life expectancy than spermidine concentration (Binh et al., 2010; Senekowitsch et al., 2023). These observations may reflect the state of quasi‐equilibrium between spermidine and spermine blood concentrations, as reflected in their strong positive correlation in our data. Whatever the source of spermidine, our data strongly support a genetic contribution to erythrocyte concentrations of spermidine and a range of biochemically associated molecules.

Existing work on spermidine is consistent with the novel MCV trend we report in Figure 5. Just as MCV was discovered to deviate from expected trends in subjects experiencing extreme longevity, blood spermidine concentration has been reported to deviate similarly from age‐related trends, with nonagenarians and centenarians exhibiting higher blood levels than expected (Scalabrino & Ferioli, 1984). This phenomenon is further exemplified by metabolomic data from naked mole rats whose lifespan are ten times greater than other murine species and whose blood spermidine concentrations anomalously rise during aging (Viltard et al., 2019).

The common nonlinear trends and strong negative correlation between the spermidine molecular cluster and MCV identify a potential relationship of interest. Existing work links MCV to a host of age‐related changes and conditions. Several studies in humans have associated larger MCV with poorer cognition and performance on memory tests, even in adults as young as 20 years old (Danon et al., 1992). This association was recapitulated recently even after accounting for other demographic and health confounders and has also been demonstrated in rats (Gamaldo et al., 2011; Spangler et al., 1995). Interestingly, smaller MCV has also been linked to evolutionary advantages in other species; female bushy‐tailed woodrats prefer mates with a smaller MCV, and skuas with large MCVs tend to fledge fewer chicks (Bearhop et al., 1999; Weber et al., 2007). Associations such as these support the view of MCV as a proxy for other health metrics and overall well‐being.

Higher MCVs have been associated with increased infectious complications following surgery and higher mortality risk in cancer patients and healthy populations (Jomrich et al., 2019; Lam et al., 2013; Nagai et al., 2018; Sakamoto et al., 2021; Yoon et al., 2016; Zheng et al., 2013). Although factors such as nutrition, chemotherapy, and oxidative stress have been implicated in these observations, a pathologic mechanism is not clearly understood and does not explain the predictive value of higher MCVs in healthy adults (Yoon et al., 2016). In several studies, increased mortality risk is observed at upper ranges of MCV well within the normal range. In interpreting the relationship between MCV and risk of mortality, it must be emphasized that in adults 40%–55% of variability in MCV is heritable, but in a very large study of 12 year‐old twins, MCV had a heritability estimate of 96% (Evans et al., 1999). These two observations indicate a high degree of heritability of MCV modified by a significant impact of lifestyle, environment, and aging. Therefore, among all effects, the undeniable heritability of MCV and the associated complex biochemical phenotype encompassing energy metabolism, redox homeostasis, and autophagy constitutes a plausible phenotypic marker of mortality risk and longevity.

Beyond previously identified cognitive changes, we further connected MCV to aging by revealing that those who experience extreme longevity possess significantly smaller MCVs than expected for their age. MCV is posited as a marker of overall erythrocyte health and robustness because it represents average cell age. Since erythrocytes decrease in size as they age (Shperling & Danon, 1990), a smaller MCV indicates that—on average—red blood cells are surviving in circulation for a longer period of time, potentially because they are more robust to stress and more effective at cellular housekeeping. These observations, combined with the consistent negative correlation between MCV and the cluster of longevity‐associated molecules, suggest that MCV may be a convenient marker of biological variability in overall body cell robustness.

This potential utility as a marker of health and robustness is further strengthened in our work and others by a direct link between MCV and rate of all‐cause mortality (Yoon et al., 2016). It is hypothesized that the same factors that shorten erythrocyte lifespan may contribute to decreased overall life expectancy, and that longevity in subjects with smaller MCVs than expected for their age is partly because they are better able to manage cellular housekeeping pathways and forestall major causes of mortality such as cancer, cardiovascular disease, and neurodegenerative disease. An abundance of experimental data on spermidine demonstrates this effect on longevity.

Whether the erythrocyte phenotype described in this study plays an active role in longevity remains to be determined. But the elements of the phenotype—energy metabolism, redox homeostasis, and autophagy—can be plausibly related to erythrocyte lifespan and development. Individuals inheriting erythrocytes with higher capacities for energy metabolism and redox homeostasis might be expected to have longer erythrocyte lifespans, and therefore smaller MCVs. Additionally, the observed higher concentrations of autophagy‐related molecules might reflect a more robust process of autophagy during erythropoiesis, which is undoubtedly a massively consequential process as erythroblasts transform into mature erythrocytes. Whether these inherited biological variables are unique to erythrocytes or reflect a more generalized biochemical phenotype in the human body are important targets of future studies.

In addition to providing insight into the biological mechanisms of aging, identification of biomarkers of healthy aging, as represented in this study, may enable early identification of populations or individuals at increased risk of age‐related disease. Perhaps more importantly, identifying the biological underpinnings of this phenomena may lead to future interventions that support better health and longer life.

## AUTHOR CONTRIBUTIONS

C.J.K., J.M., A.H., and T.R. conceptualized and designed the study. C.J.K., J.M., A.H., A.S.H., and T.R. analyzed the data. C.J.K. and J.M. prepared the first draft of the manuscript. All authors collected, assembled, and interpreted the data and helped configure and revised the manuscript.

## FUNDING INFORMATION

This research was supported in part by R01 AG052324 (to L.L.) and R01AG078794 (to L.L.).

## CONFLICT OF INTEREST STATEMENT

The authors declare no competing interests.

## Supporting information


Table S1.

## Data Availability

The metabolomics data that support the findings of this study are openly available in Metabolomics Workbench at https://doi.org/10.21228/M8KB0M, project ID PR001912. Mean Cell Volume dataset is openly available through Harvard Dataverse https://doi.org/10.7910/DVN/NXTUOS “Erythrocyte Mean Cell Volume vs Living Status”. Proteomics data are openly available through the Center for Computational Mass Spectrometry MassIVE https://doi.org/10.25345/C5JW86Z5X ID MSV000093812. Processed data containing heritability estimates for both proteins and metabolites as well as the corresponding twin pair data are openly available through Harvard Dataverse https://doi.org/10.7910/DVN/YPBN3H “RBC Twin Study Metabolite and Protein Heritability Raw and Processed Data”.
