# Supplementary material for: The longevity factor spermidine is part of a highly heritable complex erythrocyte phenotype associated with longevity
Source: Aging Cell. 2024 Sep 7;23(12):e14311. doi: 10.1111/acel.14311 (PMC11634715; doi:10.1111/acel.14311)
Supplement: Supplementary file 1 — Table S1. [file ACEL-23-e14311-s001.pdf]

Supplemental Table 1. Metabolites significantly correlated with spermidine.

Molecule	Pathway	Pearson correlation coefficient	p-value	Heritability
Spermine	Polyamine metabolism	0.736	3.22E-07	N/A
Mannose-6-phosphate	Fructose, mannose and galactose metabolism	0.678	5.58E-06	64.1
2,3-diphosphoglycerate	Glycolysis, gluconeogenesis, and pyruvate metabolism	0.666	9.18E-06	84.8
3-phosphoglycerate	Glycolysis, gluconeogenesis, and pyruvate metabolism	0.637	2.94E-05	75.4
Acetylcarnitine	Fatty acid metabolism (acyl carnitine)	0.595	1.29E-04	<20
Stearate (18:0)	Long chain fatty acid	0.593	1.39E-04	54
Taurine	Methionine, cysteine, SAM and taurine metabolism	0.560	3.79E-04	78.7
Carnitine	Carnitine metabolism	0.559	3.96E-04	<20
S-methylglutathione	Glutathione metabolism	0.555	4.48E-04	<20
Phosphoenolpyruvate (PEP)	Glycolysis, gluconeogenesis, and pyruvate metabolism	0.552	4.80E-04	77.7
Maltotriose	Glycogen metabolism	0.550	5.09E-04	<20
Maltotetraose	Glycogen metabolism	0.547	5.50E-04	<20
Ethanolamine	Phospholipid metabolism	0.539	6.96E-04	82.7
Nicotinamide	Nicotinate and nicotinamide metabolism	0.538	7.16E-04	38.1
S-lactoylglutathione	Glutathione metabolism	0.502	0.0018	62.8
N-acetylmannosamine	Aminosugar metabolism	0.499	0.0019	67.9
2-hydroxyglutarate	Fatty acid, dicarboxylate	0.481	0.0030	N/A
Margarate (17:0)	Long chain fatty acid	0.480	0.0030	43.4
Isobutyrylcarnitine	Isoleucine, leucine, and valine metabolism	0.479	0.0031	79.2
Adenosine	Purine metabolism, adenine containing	0.456	0.0052	77.5

Succinylcarnitine	TCA cycle	0.454	0.0054	48.2
Ophthalmate	Glutathione metabolism	0.454	0.0055	40.7
Threonine	Glycine, serine, and threonine metabolism	0.453	0.0056	64.4
Tetradecanedioate	Fatty acid, dicarboxylate	0.451	0.0058	26.3
Guanosine 5'-diphospho-fucose	Purine metabolism, guanine containing	0.448	0.0061	75.02
Pyruvate	Glycolysis, gluconeogenesis, and pyruvate metabolism	0.447	0.0063	60.8
2-methylbutyrylcarnitine (C5)	Isoleucine, leucine and valine metabolism	0.446	0.0065	<20
3-hydroxybutyrate (BHBA)	Ketone bodies	0.446	0.0065	<20
Glutathione, reduced (GSH)	Glutathione metabolism	0.445	0.0065	55.8
Propionylcarnitine	Fatty acid metabolism; BCAA metabolism	0.433	0.0084	71.3
Hypotaurine	Methionine, cysteine, SAM and taurine metabolism	0.429	0.0090	49.6
Glycerol 3-phosphate (G3P)	Glycerolipid metabolism	0.416	0.0115	59
Pentulose 5-phosphate	Pentose phosphate pathway	0.415	0.0119	55.5
Dihydroxyacetone phosphate (DHAP)	Glycolysis, gluconeogenesis, and pyruvate metabolism	0.415	0.0119	78.8
Palmitate (16:0)	Long chain fatty acid	0.401	0.0153	<20
Dodecanedioate	Fatty acid, dicarboxylate	0.398	0.0162	N/A
Kynurenine	Tryptophan metabolism	0.395	0.0171	<20
Adrenate (22:4n6)	Polyunsaturated fatty acid (n3 and n6)	0.391	0.0183	<20
Adenosine 5'-monophosphate (AMP)	Purine metabolism, adenine containing	0.388	0.0193	72.5
Glycine	Glycine, serine and threonine metabolism	0.387	0.0196	66.1
6-phosphogluconate	Pentose phosphate pathway	0.379	0.0226	N/A
3-dehydrocarnitine	Carnitine metabolism	0.374	0.0247	60.7
Dihomo-linoleate (20:2n6)	Polyunsaturated fatty acid (n3 and n6)	0.371	0.0260	37.7

Myo-inositol	Inositol metabolism	0.361	0.0303	39
Glutathione, oxidized (GSSG)	Glutathione metabolism	0.360	0.0310	71.4
Maltose	Glycogen metabolism	0.359	0.0317	50.7
Nonadecanoate (19:0)	Long chain fatty acid	0.358	0.0318	<20
S-methylcysteine	Methionine, cysteine, SAM and taurine metabolism	0.343	0.0403	21.8
Glutamate	Glutamate metabolism	0.338	0.0440	90.9
Linoleate (18:2n6)	Polyunsaturated fatty acid (n3 and n6)	0.337	0.0445	40.7
Heme	Hemoglobin and porphyrin metabolism	0.332	0.0477	56.6
Threonate	Ascorbate and aldarate metabolism	-0.355	0.0338	72.4
Valylhistidine	Dipeptide	-0.361	0.0307	71.1
Pantothenate	Panthothenate and CoA metabolism	-0.371	0.0260	<20
1-stearoylglycerophosphoserine	Lysolipid	-0.383	0.0211	78.9
1-oleoylglycerophosphocholine (18:1)	Lysolipid	-0.396	0.0168	N/A
Caproate (6:0)	Medium chain fatty acid	-0.449	0.0060	N/A
N-acetylphenylalanine	Phenylalanine and tyrosine metabolism	-0.519	0.0012	N/A
Caprylate (8:0)	Medium chain fatty acid	-0.528	0.0009	N/A

Supplemental Table 2. Proteins significantly correlated with spermidine.

Molecule	Pearson correlation coefficient	p-value	Heritability
NSF	0.627	4.32E-05	32.4
P4HB	0.579	0.00021	63.59
GSR	0.578	0.00022	<20
PDCD6IP	0.531	0.00087	46.28
STX7	0.524	0.00105	59.7
EIF2S1	0.521	0.00113	81.6
SACM1L	0.488	0.00255	22.93
EIF2S3; EIF2S3L	0.485	0.00272	47.25
EHD1	0.470	0.00386	76.25
GANAB	0.470	0.00383	51.58
YARS	0.469	0.00393	63.85
RNH1	0.465	0.00427	41.19
HNRNPK	0.445	0.00654	50.7
XPO7	0.435	0.00804	<20
ABCC4	0.430	0.00894	81.31
CHMP4A	0.425	0.00983	<20
PA2G4	0.425	0.00975	46.85
DIAPH1	0.419	0.01090	<20
HSPA5	0.409	0.01322	<20
ADD3	0.404	0.01465	81.25
TPM1	0.393	0.01786	20.73
DNM2	0.385	0.02038	50.32
TUBB4B; TUBB; TUBB2B; TUBB2A; TUBB4A; TUBB3	0.379	0.02245	<20

S100A9	0.366	0.02825	82.26
BLVRB	-0.369	0.02657	<20
PGAM1; PGAM2	-0.385	0.02041	29.5
HAGH	-0.394	0.01727	<20
